# Functional studies of BCL11A: characterization of the conserved BCL11A-XL splice variant and its interaction with BCL6 in nuclear paraspeckles of germinal center B cells

**DOI:** 10.1186/1476-4598-5-18

**Published:** 2006-05-16

**Authors:** Hui Liu, Gregory C Ippolito, Jason K Wall, Teresa Niu, Loren Probst, Baeck-Seung Lee, Karen Pulford, Alison H Banham, Luke Stockwin, Arthur L Shaffer, Louis M Staudt, Chhaya Das, Martin JS Dyer, Philip W Tucker

**Affiliations:** 1 Section of Molecular Genetics and Microbiology and Institute for Cellular and Molecular Biology, 1 University Station, A5000, University of Texas, Austin, Texas, 78712, USA; 2 Nuffield Department of Clinical Laboratory Sciences, Room 4A10, Level 4 Academic Block, John Radcliffe Hospital, Oxford, OX3 9DU, UK; 3 Metabolism Branch, Division of Clinical Sciences, Building 10, Room 4N114, National Cancer Institute, Bethesda, MD, 20892, USA; 4 MRC Toxicology Unit, University of Leicester, PO Box 138, Lancaster Road, Leicester LE1 9HN, UK

## Abstract

**Background:**

Chromosomal aberrations of *BCL11A *at 2p16.1 have been reported in a variety of B-cell malignancies and its deficiency in mice leads to a profound block in B-cell development.

**Results:**

Alternative pre-mRNA splicing of *BCL11A *produces multiple isoforms sharing a common N-terminus. The most abundant isoform we have identified in human lymphoid samples is *BCL11A-XL*, the longest transcript produced at this locus, and here we report the conservation of this major isoform and its functional characterization. We show that BCL11A-XL is a DNA-sequence-specific transcriptional repressor that associates with itself and with other BCL11A isoforms, as well as with the BCL6 proto-oncogene. Western blot data for BCL11A-XL expression coupled with data previously published for BCL6 indicates that these genes are expressed abundantly in germinal-center-derived B cells but that expression is extinguished upon terminal differentiation to the plasma cell stage. Although BCL11A-XL/BCL6 interaction can modulate BCL6 DNA binding *in vitro*, their heteromeric association does not alter the homomeric transcriptional properties of either on model reporter activity. BCL11A-XL partitions into the nuclear matrix and colocalizes with BCL6 in nuclear paraspeckles.

**Conclusion:**

We propose that the conserved N-terminus of BCL11A defines a superfamily of C2HC zinc-finger transcription factors involved in hematopoietic malignancies.

## Introduction

Malignancies of mature B lymphocytes are characterized by chromosomal translocations involving the immunoglobulin heavy chain (*IGH*) locus on chromosome 14q32.3, resulting in the deregulated expression of the translocated proto-oncogene. Although t(2;14)(p16.1;q32.3) is a rare event in B cell malignancies, gains and amplifications of 2p16.1 (previously mapped as 2p13) have been reported in approximately 20%–50% among subtypes of diffuse large B-cell lymphoma (DLBCL) and in 50% of classical Hodgkin's lymphoma (HL) [1,2]. Previously we identified a Krüppel zinc finger-encoding gene, B-cell lymphoma/leukemia 11A (*BCL11A*) and showed it to be disrupted and deregulated in four aggressive cases of B-cell chronic lymphocytic leukemia (B-CLL) with t(2;14)(p16.1;q32.3) that lacked mutations within their expressed variable (V_H_) region genes [3-5]. The mouse orthologue, *Evi9*, was identified as a proto-oncogene by virtue of nearby recurrent retroviral insertions that correlated with the development of myeloid leukemias or B-cell lymphomas [6,7].

**Figure 1:**
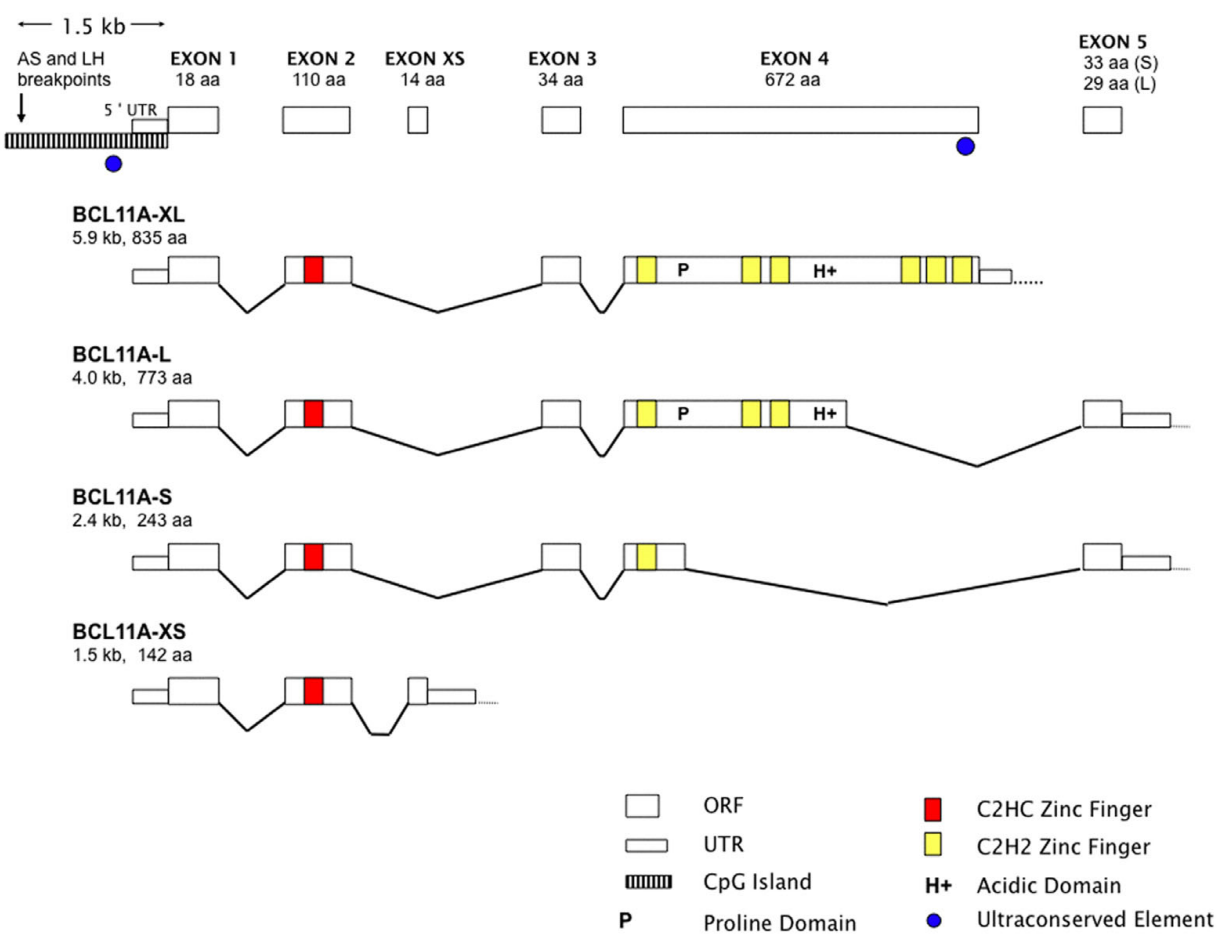
**Human BCL11A locus and predicted protein isoforms**. Alternative splicing within the *BCL11A *locus (Entrez Gene ID 53335) leads to the creation of four major protein isoforms: eXtra Long (XL; accession AJ404611), Long (L; accession AJ404612), Short (S; accession AJ404613), and eXtra Short (XS; accession AY692278). Exons 1 and 2 are common to all four isoforms, which encode a superfamily repression domain and also an unusual C2HC zinc finger likely to be involved in protein-protein interactions (see Discussion). Unlike the XS isoform, XL, L and S all employ at least a portion of Exon 4 (which encodes all six Krüppel-type C2H2 zinc fingers). Also shown are the chromosomal breakpoints [3], 1.5 kb upstream of Exon 1, that first were described in pediatric B-CLL patients AS and LH who bore the reciprocal translocation t(2;14) (p16.1;q32.3).

Alternative pre-mRNA splicing of human *BCL11A *(Figure 1) leads to a minimum of four transcripts predicted to yield protein isoforms designated as eXtra-Long (XL; 5.9 kb/125 kD), Long (L; 3.8 kb/100 kD), Short (S, 2.4 kb/35 kD) and eXtra-Short (XS, 1.5 kb/25 kD). Exons 1 and 2 are common to all isoforms, whereas XL, L and S each utilize at least a portion of exon 4, leading to a variable number of C2H2 zinc fingers appended to the invariant C2HC zinc finger at the N-terminus. *BCL11A-XL *RNA is expressed at high levels in normal as well as malignant lymphoid tissues, including germinal center (GC) B cells, B-CLL, follicular lymphoma (FL), and DLBCL [4,8,9]. Furthermore, aside from its predominant expression within the B-cell compartment, high levels of *BCL11A-XL *RNA also accumulate in both fetal and adult brain, and in the plasmacytoid dendritic cell [4,8].

Although less thoroughly studied, *Bcl11a/Evi9 *transcript expression in the mouse mirrors that seen in the human, with highest accumulation in brain and B lymphoid cells [8]. B-cell development in Evi9 null mice generated by conventional targeted disruption is blocked at the earliest progenitor B-cell stage, underscoring the essential nature of this locus in the immune system [10]. The mouse equivalent of the human *BCL11A-L *isoform has been shown to interact and colocalize with BCL6, another Krüppel zinc-finger proto-oncogene expressed highly in the germinal center and frequently deregulated by translocations in FL and DLBCL [1,7]. However, no functional consequences of BCL11A-BCL6 colocalization have been reported. Putative transactivation and dimerization regions as well as a potential preferred DNA binding site have been published for the murine L form [11].

The mouse *Evi9 *locus is organized quite similarly, and the genes are extraordinarily conserved at nucleotide (94%) and amino acid (95%) levels, placing *BCL11A/Evi9 *within a small subset of "ultra-conserved" genes [12]. Nonetheless, a murine counterpart of the XL isoform has not been previously described [7,11,13]. Here, we document the conservation of *BCL11A-XL *in mouse (and chicken) and we report the first functional analysis of the human BCL11A family, with emphasis on the highly expressed XL isoform. This isoform, as with all known BCL11A isoforms, has an N-terminus that encodes a distinctive 12-amino-acid peptide as well as an atypical C2HC zinc finger; these two protein motifs are stringently conserved among a variety of hematopoietic transcription factors and allow the definition of a new superfamily.

## Results

### BCL11A-XL protein accumulates highly within the nuclear matrix of germinal-center lymphomas and B-cell lines and is evolutionarily conserved

Previous studies have documented elevated RNA levels of *BCL11A *in GC-derived lymphomas such as B-CLL, FL, and Burkitt's lymphoma [1,4,9]. Conversely, post-GC lymphomas, regardless of the cytogenetic status of 2p16.1, express significantly lower RNA levels of *BCL11A *isoforms (unpublished observations). We tested whether this differential pattern of RNA expression of BCL11A extends to the protein level. We generated the BCL11A/123 monoclonal antibody [14] specific to the region containing zinc fingers 4–6 which is fully retained in BCL11A-XL, partially shared by BCL11A-L, but excluded by alternative splicing in BCL11A-S and BCL11A-XS (Figure 1).

**Figure 2:**
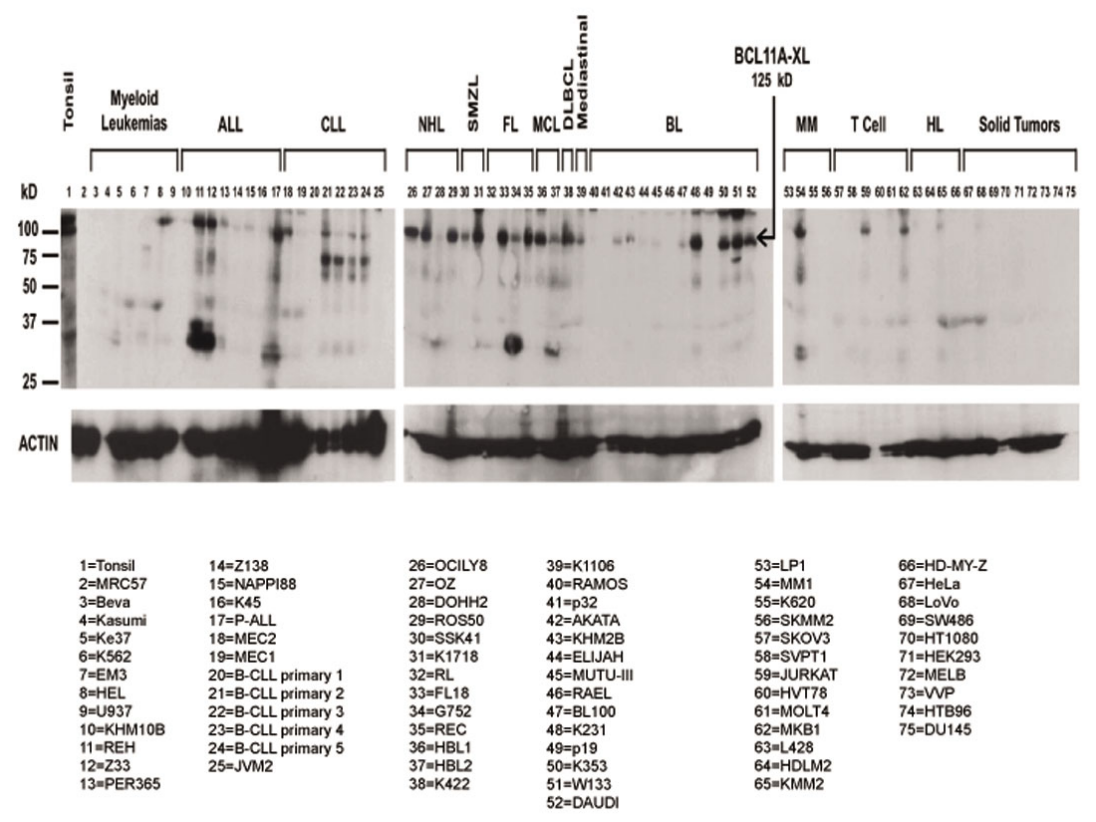
**The XL isoform of BCL11A is highly expressed in a variety of pre-GC and GC-derived B-cell malignancies**. Western blotting using BCL11A/123 to detect BCL11A-XL in a panel of malignant lymphoid cell lines. Cell types, indicated at the top with individual representatives (lane numbers) are arrayed from left to right according to B-cell development status. ALL, B-cell acute lymphocytic leukemia; CLL, B-cell chronic lymphocytic leukemia; NHL, non-Hodgkin's lymphoma; SMZL, small marginal zone lymphoma; FL, follicular lymphoma; MCL, Mantle cell lymphoma; DLBCL, diffuse large B cell lymphoma; BL, Burkitt's lymphoma; MM, multiple myeloma; HL, Hodgkin's lymphoma. Expression is highest in samples representative of earlier stages of B-cell differentiation (e.g., CLL, FL). Expression is low to negative in cell lines representative of later (post-germinal-center) stages of differentiation (e.g., MM and HL). The filter was stripped and re-probed (bottom panel) with anti-β-actin as a loading control.

BCL11A/123 detects endogenous expression of BCL11A-XL in human B-cell lines (Figure 2) previously documented to express high levels of *BCL11A *RNA transcripts. Its size (~125 kD) is indistinguishable from that observed in vitro or when transfected (see below). As a trend, XL protein expression prevailed among pre-GC and GC-derived B-lymphoid cell lines, but was low to negative in cell lines representative of late-GC or post-GC stages of B-cell development. Among the post-GC lines, the majority of multiple myeloma (MM) and Hodgkin's lymphoma (HL) samples were negative for BCL11A-XL. Lastly, BCL11A was not expressed in the majority of myeloid and T-cell leukemias.

**Figure 3:**
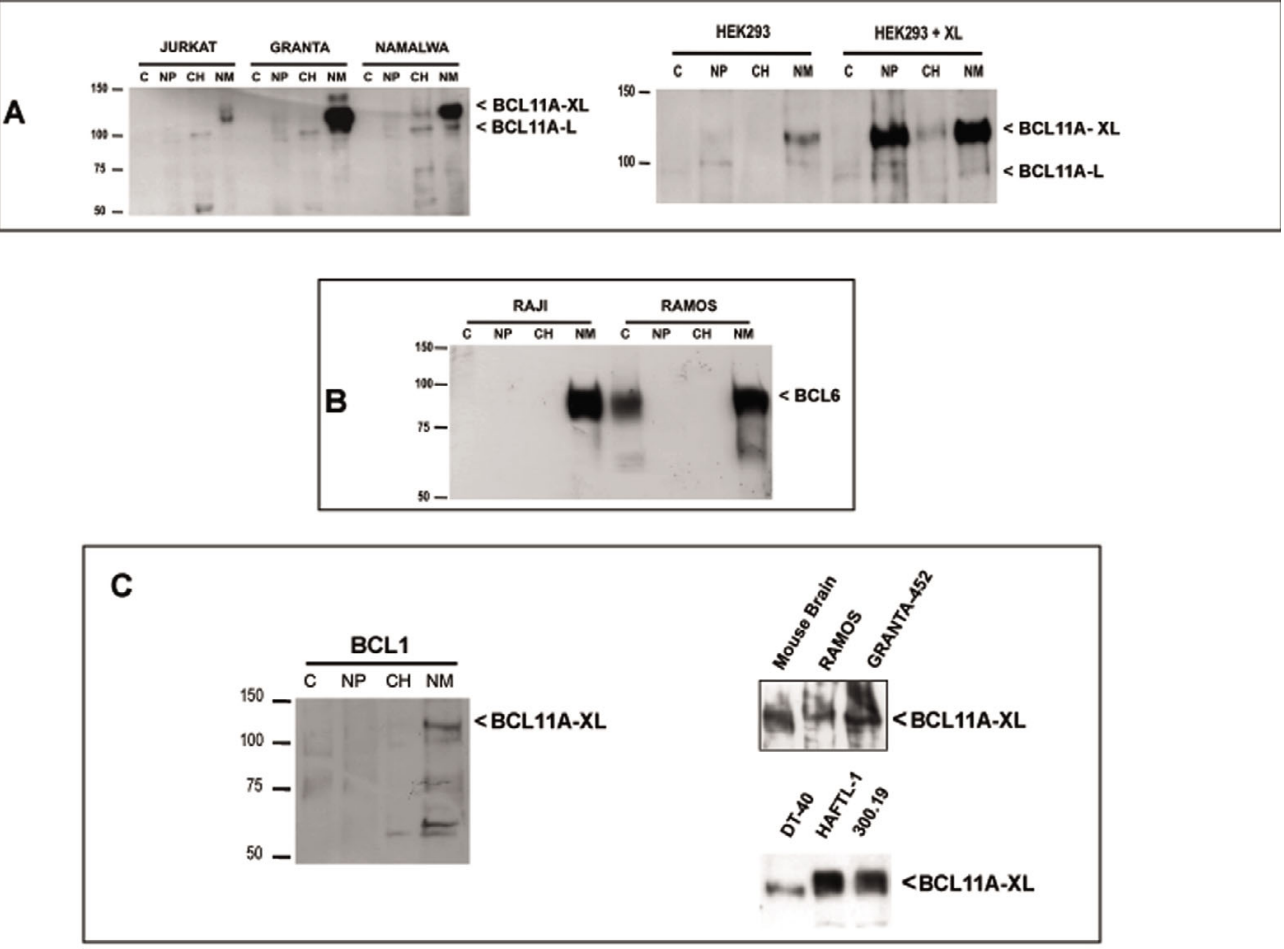
**BCL11A-XL protein accumulates in the nuclear matrix of pre-germinal center lymphomas and B cell lines**. (**A**) BCL11A-XL, whether endogenous (left panel) or ectopically expressed as an N-terminal FLAG-tagged fusion in HEK293 cells (right panel) partitions predominantly into the nuclear matrix fraction. Cells were fractionated using the method of Reyes et al [15], and equivalent volumes of each fraction were loaded per lane, allowing us to assess the relative distribution by western blotting with BCL11A/123. Cellular compartments were fractionated as Cytoplasm (C), Nucleoplasm (NP), Chromatin (CH), and Nuclear Matrix (NM). BCL11A-L partitions predominantly into the chromatin-associated fraction. BCL11A-S is predominantly cytoplasmic (data not shown). Note that HEK293 cells express endogenous XL protein in the NM fraction. (**B**) Endogenous BCL6, like BCL11A-XL, accumulates within the nuclear matrix. **(C) **Conservation of the XL isoform is documented by Western blotting a murine B-cell line (BCL1), mouse whole brain tissue, and the chicken DT40 B-cell line.

We fractionated lysates prepared from the Granta-452 and Namalwa lymphoid-derived cell lines expressing high levels of BCL11A-XL into cytoplasm (C), nucleoplasm (NP), chromatin (CH), and nuclear matrix (NM). Western blotting studies of lymphoid cell lines (Figure 3A) demonstrate that the XL isoform is sequestered mostly into the NM fraction. The L form can localize additionally into the CH. Endogenous BCL11A expression in HEK293 cells is moderate compared to B-lymphoid cell lines and shows a similar enrichment of XL in the NM fraction, although when transfected, ectopic BCL11A-XL in HEK293 localizes to the NP fraction as well (Figure 3A). We further observed that BCL6 fractionated primarily into the NM regardless of whether endogenously or exogenously expressed (Figure 3B). Curiously, although absent in the electronic databases and undocumented in the literature, we easily detected BCL11A-XL in tissues and cell lines derived from mouse and chicken (Figure 3C).

### BCL11A-XL interacts with BCL6, with itself, and with the other BCL11A isoforms

**Figure 4:**
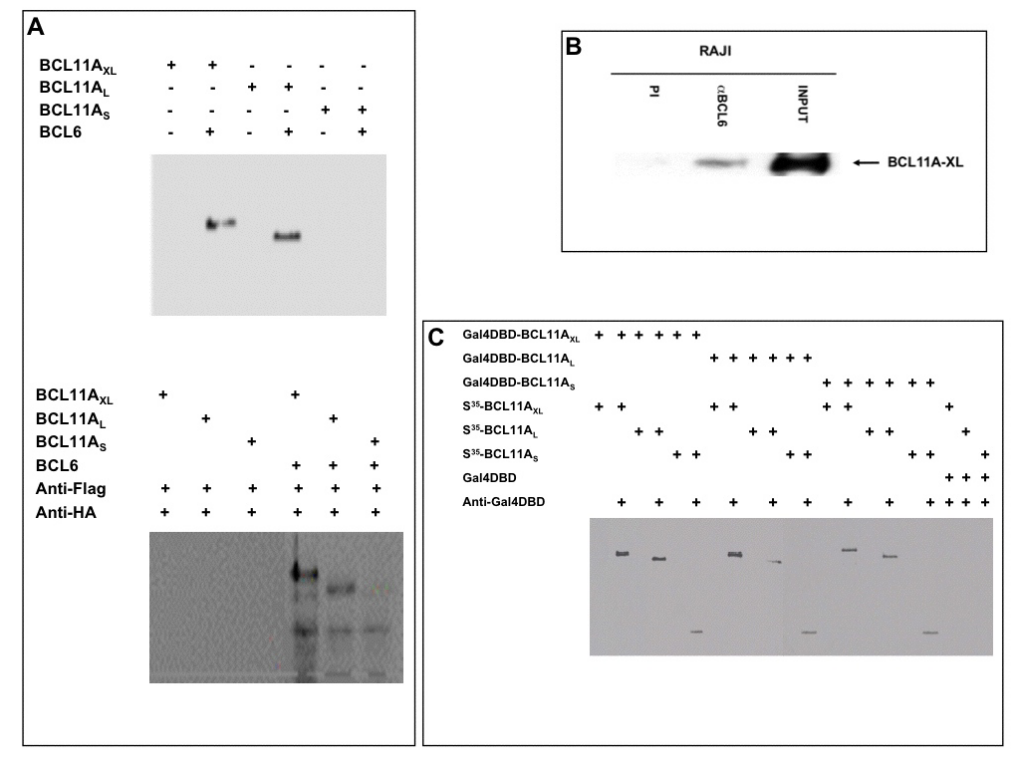
**Interaction of BCL11A-XL with BCL6, with itself, and with other BCL11A isoforms**. (**A**) XL and L, but not S, associate with BCL6. Top, *in vitro *translated 35S-labeled BCL11A isoforms were incubated with cell lysates of HEK293 cells transiently transfected with FLAG-BCL6. Complexes were immunoprecipitated with anti-FLAG, separated on SDS-PAGE, and then visualized by fluorography. Bottom HEK293 cells were transiently cotransfected with FLAG-BCL6 and HA-BCL11A isoform constructs. 48 hrs post-transfection, cell lysates were prepared, immunoprecipitates were prepared with anti-FLAG and products resolved as above were detected by anti-HA Western blotting. (**B**) Identification of endogenous BCL11A-XL and BCL6 heteromeric complexes in B-cell lines. Whole cell lysates from the Burkitt's lymphoma cell line, Raji, were immunoprecipitated with anti-BCL6 polyclonal antisera, resolved on SDS-PAGE and blotted for BCL11A-XL. (**C**) All BCL11A isoforms interact with each other *in vitro*. Immunoprecipitations were performed using *in vitro *translated, unlabeled Gal4-DNA-binding domain (Gal4DBD) alone or Gal4DBD-BCL11A isoform fusion proteins, and *in vitro *translated 35S-labeled HA-BCL11A isoforms, in the presence or the absence of an anti-Gal4-DNA-binding domain antibody (anti-Gal4DBD). The immunoprecipitated products were separated on SDS-PAGE and visualized by fluorography.

Previous studies showed that the mouse *Evi9a *L-form colocalized and associated with BCL6 when ectopically over-expressed in non-B cells [7]. To test this for the human isoforms, we first performed co-immunoprecipitation assays using proteins generated *in vitro *and in transient co-transfections of COS-7 or HEK293 cells. As expected, human BCL11A-L interacted with BCL6 in both assays. No interaction between BCL11A-S and BCL6 was detected (Figure 4A). XL interacted strongly with BCL6 under these artificial conditions, but importantly, the endogenous complex could also be immunoprecipitated from a B-cell line (Figure 4B). A similar series of immunoprecipitations revealed the surprising result that all three isoforms can interact with each other or with themselves (Figure 4C). These data indicate that interaction faces essential for homomeric interactions (the exons common to all isoforms) differ from those essential for heteromeric association with BCL6 (the exons not shared with the S isoform). Further, they raise the possibility that the relative ratios of BCL6 and the BCL11A isoforms may determine their DNA binding or transcriptional regulatory properties.

### BCL11A-XL and BCL6 interactions affect respective DNA binding activities in vitro but have little effect on respective transcriptional repression activities in vivo

**Figure 5:**
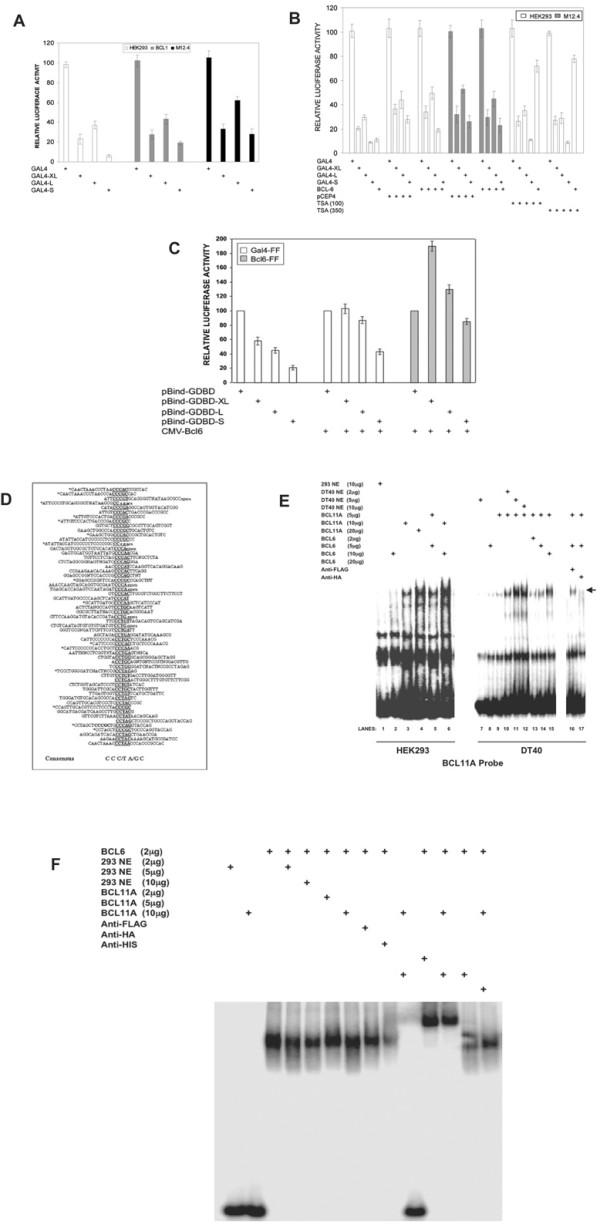
**Transcriptional and DNA binding effects of BCL11A homomeric and BCL11A-BCL6 heteromeric complexes on artificial target genes**. (**A**) Gal4DBD-BCL11A isoforms repress Gal4-mediated Firefly luciferase activity of pG5luc in BCL1 and M12.4 B cells or in HEK293 cells. Dual luciferase assays were performed 48 hr post-transfection into the indicated cell lines on whole cell lysates shown by Western blotting to contain approximately equal amounts of Gal4-BCL11A isoforms (data not shown). Values are the average of three independent experiments and are expressed as percent of those obtained using the reporter construct and Gal4DBD alone after normalization of transfection efficiency for the activity of Renilla luciferase. (**B**) Repression by BCL6, but not BCL11A, is TSA sensitive. pG5*luc *and Gal4DBD-BCL11A isoform fusions or FLAG-BCL6 along with a *firefly *luciferase reporter vector containing 5×-BCL6 binding sites upstream of the minimal SV40 promoter (5XBCL6-SV40-Luc; REF), and a *Renilla *luciferase control plasmid were transiently cotransfected into HEK293 cells. Indicated amounts of TSA were added into the cultures 24 hr after transfection, then 24 hr later Luciferase activity was measured. The values represent the average of three independent experiments. BCL6 does not affect BCL11A isoform repression in HEK293 and M12.4 cells when expressed at levels sufficient for co-immunoprecipitation. Transient co-transfections were carried out in triplicate, normalized and plotted as described above with pG5*luc *luciferase and Gal4DBD-BCL11A isoform fusions with or without FLAG-BCL6 or an irrelevant DNA (pCEP4) to equalize input DNA. (**C**) Reciprocal de-repression of BCL6 and BCL11A-XL in chicken DT-40 B cells. Co-transfection of FLAG-BCL6 decreases Gal4-mediated luciferase (GAL4-FF) repression of Gal4-BCL11A-XL isoforms. Co-transfection of HA-tagged BCL11A isoforms releases site-specific repression on 5XBCL6-SV40-Luc. Cells were transfected by electroporation and dual luciferase activities measured and normalized as above. (**D**) Identification of a specific DNA binding motif for BCL11A-XL. Cyclic amplification of targets (CASTing) for consensus sequences. *In vitro *translated N-terminal HA-tagged BCL11A-XL was subjected to 5 rounds of binding/PCR with a random 17-mer bridged by vector/cloning sites, as achieved by immobilization and immunoprecipitation with an anti-HA mAB and protein A beads. Cloned and sequenced candidates are aligned, with lower case letters denoting vector sequence and asterisks denoting sequences that contain >1 putative consensus (indicated at the bottom). (**E**) BCL6-BCL11A association results in enhanced binding of BCL11A-XL to it target sequence. Left, EMSA performed with the BCL11A consensus oligonucleotide probe (4× CASTing sites as determined in [D]) using the indicated protein extract amounts (μg) prepared from single transfections of FLAG-BCL6 or HA-BCL11A-XL into HEK293 cells. Right, EMSA with same probe, utilizing tagged-proteins from transfected DT40 B cells. The remaining HA-BCL11A-XL/DNA complex is ablated by anti-HA but not affected by anti-FLAG, indicating that no heteromeric complex is formed. (**F**) BCL6/BCL11A-XL association results in subtly decreased binding of BCL6 to specific DNA target sequence. The tandem BCL6 binding site repeat was isolated as a fragment from 5XBCL6-SV40-Luc, labeled with 32P and used as a probe in EMSA (detailed in Methods and Materials). The indicated amounts (μg) of proteins, prepared from nuclear extracts of untransfected HEK293 cells or BCL11A-XL transfected HEK293, and antibodies used in super-shifts were added (+) or not (-) to a constant amount (2 μg) of FLAG-BCL6 prepared in the same manner.

Initially we tested whether N-terminal Gal4-DNA-binding domain (Gal4DBD) fusions of XL and the other BCL11A isoforms could modulate transcriptional activity of a firefly reporter bearing 4x-Gal4 DNA binding sites. Results of co-transfections in HEK293 cells (BCL11A-positive; see Figure 3A) and in the murine B-cell lines BCL1 (BCL11A-positive, Figure 3C) and M12.4 (BCL11A-negative, data not shown) are shown in Figure 5A. All fusion BCL11A constructs significantly repressed transcription 5–10 fold in both cell types. Unexpectedly, the predominantly cytoplasmic S isoform consistently showed higher levels of repression than XL or L. Most of BCL11A-S was artificially driven into the nucleus by the strong nuclear localization sequence (NLS) present on the Gal4DBD (data not shown). No significant alteration of BCL11A activity was observed in the presence of the histone deacetylase inhibitor, trichostatin A (TSA), although consistent with previous findings, BCL6 repression was released up to 7-fold (Figure 5B). These results indicate that homomeric BCL6 and BCL11A execute trans-repression by different mechanisms.

Although BCL11A-XL and L interact with each other and with BCL6 (Figure 4A and 4B), we observed no effect of BCL6 on BCL11A repression in co-transfected cells (Figure 5B). Similarly, we observed no synergistic or even additive effects when various BCL11A isoforms were co-transfected (data not shown). Since BCL6 has been shown to repress by binding to a specific consensus motif within target genes [17], we employed a luciferase reporter in which the minimal SV40 promoter is appended upstream with 5×-BCL6 binding sites in tandem. Neither BCL11A-XL nor the other isoforms significantly affected BCL6-mediated repression in HEK293 cells (data not shown). When these experiments were performed in DT40 chicken-derived pre-B cells, a modest yet consistent de-repression was reciprocally observed (Figure 5C). The specificity of this effect might be questioned by the fact that the non-BCL6-interacting S isoform de-repressed to an equivalent extent. However, since all BCL11A isoforms interact with each other (Figure 4C), the relatively high endogenous levels of XL in DT40 (Figure 3C) might drive an S-XL heterodimer into the nucleus (re-addressed below and in Figure 7) where, as shown by the GAL4-fusion data of Figure 5A, its repression activity is strong.

**Figure 6:**
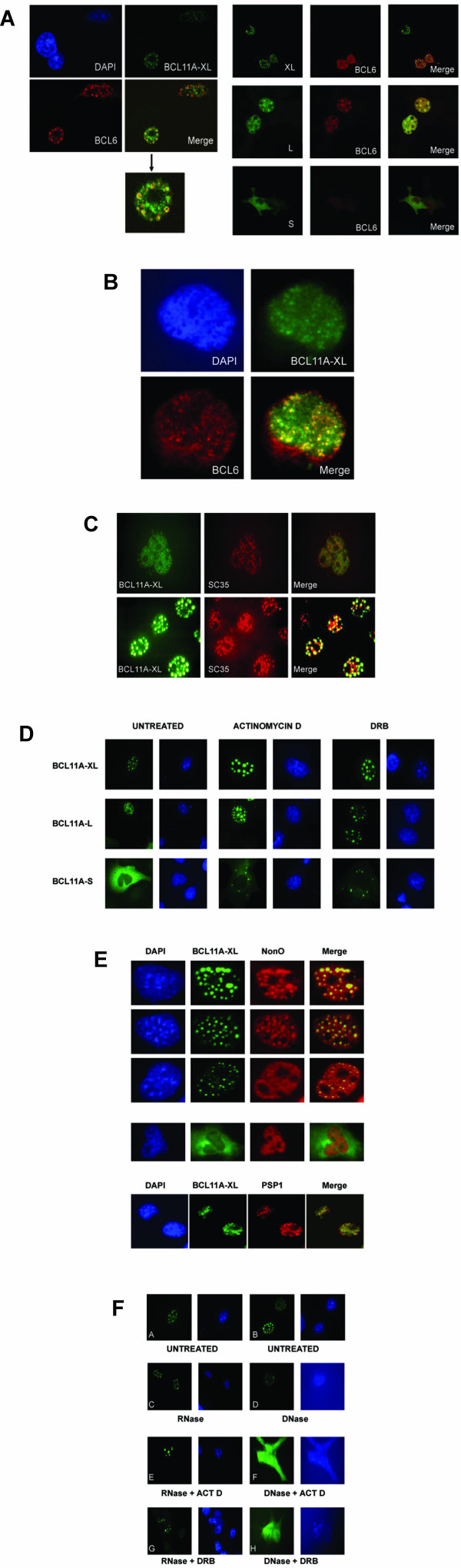
**Dynamic colocalization of BCL11A-XL and BCL6 within nuclear paraspeckles**. (**A**) Colocalization of BCL11A isoforms with BCL6. Left Panel: COS-7 cells were transiently transfected with FLAG-epitope tagged BCL11A-XL and BCL6, and developed using antibodies reactive with the proteins (see Methods). Nuclei were counterstained using Hoescht 33342. Images were acquired by confocal microscopy. Right Panel: COS-7 cells were transiently transfected with GFP-BCL11A isoform fusion constructs and with a a FLAG-tagged BCL6 construct. Immunostaining was performed 24 hrs post-transfection using monoclonal anti-FLAG primary and rhodamine-conjugated rabbit-anti-mouse secondary antibodies, and then images were collected by confocal microscopy. Colocalization was determined by merging sequentially scanned images from the two channels. (**B**) Endogenously expressed BCL11A in BJAB B cells forms nuclear dots and colocalizes with BCL6 (see Methods). (**C**) BCL11A-XL resides within subnuclear domains adjacent to speckles. GFP-BCL11A-XL transfected COS-7 cells were immunostained using an anti-SC35 mAb and rhodamine-conjugated secondary. Confocal co-localization scans were performed as above. (**D**) Transcription inhibitors alter the localization of BCL11A. COS-7 cells were transiently transfected with pEGFP-BCL11A isoform constructs. At 24 hrs after transfection, Actinomycin D or 5,6-dichloro-1 – D-ribofuranosylbenzimidazole (DRB) was added to transfected cells. Cells were observed by fluorescence microscopy 6 hrs after treatment. Untreated COS-7 cells transfected with the pEGFP-BCL11A isoform constructs are shown as controls. (**E**) Identification of BCL11A-XL, nuclear dots as paraspeckles by virtue of co-staining with PSP1 and PSP2/nonO/p54nrb antibodies. Also shown in the middle panel is BCL11A-S, characteristically localizing to the cytoplasm, which unlike BCL11A-XL does not overlap with the nuclear paraspeckles. (**F**) DNase but not RNase treatment alters the localization of BCL11A. COS-7 cells were transiently transfected with pEGFP-BCL11A-XL. At 24 hrs after transfection, cells were treated with RNase (C), RNase and Actinomycin D (E), RNase and DRB (G), DNase (D), DNase and Actinomycin D (F), or DNase and DRB (H). Cells were observed via fluorescent microscopy 6 hrs after treatments. Untreated COS-7 cells transfected with pEGFP-BCL11A-XL are shown as controls (A) and (B). Hoescht 33342 staining of corresponding nuclei is shown to the right of each green fluorescence panel.

**Figure 7:**
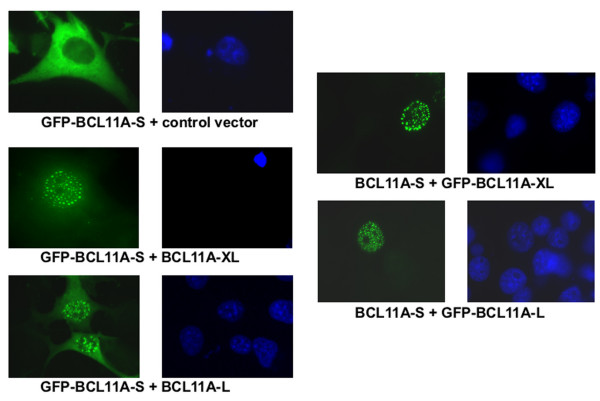
**BCL11A-S is relocalized from the cytoplasm to the nucleus by interaction with BCL11A-XL or BCL11A-L**. COS7 cells were transiently co-transfected with GFP-BCL11A-S and either the Gal4DBD as a negative interaction control (A), or with Gal4DBD-BCL11A-XL (B and D) or Gal4DBD-BCL11A-L (C and E). Cells were observed 24 hr after transfection via fluorescence microscopy. Hoescht 33342 staining of corresponding nuclei is shown to the right of each green fluorescence panel.

We employed a reiterative oligonucleotide binding and amplification approach (CASTing [16]) to determine a putative DNA binding core consensus for XL as C-C-C/T-A/G-C (Figure 5D). Although the CASTing probe produced significant non-specific binding, BCL11A-XL-transfected HEK293 cell extracts bind dose dependently (Figure 5E, lanes 3–4), whereas parent cell or BCL6-transfected extract (shown to be competent for binding its own site, Figure 5F) do not (Figure 5E, lanes 1–2). EMSAs using four repeats of this consensus as probe in transfected DT40 cell extracts showed specific binding for XL as judged by antibody super-shifts (Figure 5E, lanes 16–17). Furthermore, binding of BCL11A-XL was significantly enhanced as levels of BCL6-containing DT40 nuclear extract were increased (Figure 5E, lanes 10–12). Conversely, we observed decreased binding for BCL6/BCL11A-XL complexes on the BCL6 probe (Figure 5F).

Collectively these data allow us to conclude that the human BCL11A isoforms are capable of repressing transcription when tethered to an artificial promoter in a TSA-independent manner when ectopically and transiently over-expressed. Furthermore, BCL11A-XL appears to be capable of site-specific DNA binding. Reciprocal effects of BCL6 or BCL11A on their respective transcriptional repressive activities were modest at best, even though either protein could modulate the specific binding of the other in vitro.

### BCL11A-XL and BCL6 segregate within nuclear paraspeckles

Murine Evi9a/Bcl11a-L and BCL6 were previously reported to colocalize within an uncharacterized "nuclear dot" subdomain [7]. We observed BCL11A-XL to localize exclusively within nuclear dots, and colocalized with the major fraction of BCL6 found inside the nucleus (Figure 6A). The dot pattern for BCL11A-XL was not the result of ectopic overexpression as it was also observed in BCL11A/123 stains of endogenous XL-expressing B cell lines (Figure 6B, and data not shown). BCL11A-L, which showed fewer nuclear dots and more diffuse nuclear staining than BCL11A-XL, also appeared to colocalize with BCL6 (Figure 6A, right panel), even though at least a significant portion of L partitioned to a different (chromatin) nuclear sub-fraction biochemically (Figure 3B).

We further determined that neither the BCL11A-L nor XL-containing nuclear dots were PML or Sp100 subnuclear domains (data not shown). Although co-staining GFP-BCL11A-XL transfected cells with an antibody against SC35, a prototypic speckles marker [18], revealed a similar but non-overlapping pattern for the two proteins, GFP-BCL11A-XL appeared instead to surround the speckles and the nucleolus (Figure 6C). We also observed in other experiments that some cells could randomly display curiously large aggregates, or caps, of BCL11A-XL around the nucleolus (data not shown); further experimentation revealed that intentional blocking of transcription with chemical inhibitors could significantly increase this perinucleolar accumulation (Figure 6D). Both of these properties – adjacency to speckles and peirnucleolar relocalization – are reminiscent of the paraspeckle nuclear subdomain [18]. As shown in Figure 6E, our results show clear co-localization of BCL11A-XL with two "paraspeckle-specific" (PSP) proteins, PSP1 and nonO/p54nrb, demonstrating BCL11A-XL as a resident of the paraspeckle subnuclear body. In further support of this model, our co-immunoprecipitation experiments document protein-protein interaction (possibly indirect) between nonO and BCL11A-XL (data not shown).

BCL11A-XL and BCL6 are in the nuclear matrix (Figure 3), suggesting the possibility of cross-talk between it and the paraspeckle. Treatment of GFP-BCL11A-XL expressing cells with RNase (alone or combined with Actinomycin D) showed no significant effects on subcellular partitioning (Figure 6F). Treatment with DNase, however, led to a more diffuse perinuclear pattern, and when transcription was blocked, paraspeckles were completely disrupted (Figure 6F).

### Interaction with nuclear BCL11A isoforms can relocalize the cytoplasmic BCL11A-S to paraspeckles

BCL11A-S shows predominantly cytoplasmic localization, although small amounts of protein appear to localize diffusely within the nucleus (Figure 7). When we co-transfected GFP-BCL11A-S with GAL4-fusions of XL or L, we observed essentially quantitative translocation of S into paraspeckles, apparently indistinguishable from those of XL or L alone (Figure 7). Nuclear localization of the XL/S or L/S heteromeric complexes was dominant, in that cytoplasmic relocalization of XL or L by S was never observed (Figure 7 and data not shown). These data indicate that the relative ratios of S to XL may determine the subcellular localization of the S form, which when delivered to the nucleus is the most potent repressor (Figure 5A).

## Discussion

We have studied expression and functional features of the human BCL11A family with an emphasis on the heretofore uncharacterized XL isoform. Why XL expression has eluded detection in other species is unclear, as our data indicate that it also is a major protein isoform in mouse and chicken. Possible contributing factors might include the significant toxicity/apoptosis associated with XL over-expression (data not shown) or the general difficulties in cloning full-length mRNAs of this length (~5.9 kb), although this transcript was indeed detected but not described in a previous publication by another research group [10]. We established the expression pattern of XL and showed that it, along with S and L isoforms, can repress transcription. BCL11A-XL can bind consensus DNA target sites specifically. Finally, we showed that XL localizes to paraspeckle nuclear bodies involved in RNA metabolism, can interact with the BCL6 proto-oncogene, and can transport repression-competent, cytoplasmic BCL11A-S to the nucleus.

### BCL11A is expressed from the earliest B-cell progenitors through the GC stage, but is extinguished in terminally differentiated plasma cells

Previous studies in human or mouse were restricted to the RNA level. The transcription profile is highly similar in both species, with expression primarily in the immune and central nervous system [8,10,19]. The fact that development of Bcl11a-deficient B cells was blocked at the earliest progenitor B-cell stage [10] indicates that its expression initiates in a stem cell compartment. Microarray experiments have indicated high expression of BCL11A transcripts in CD34+ hematopoietic stem cells [8].

At the protein level, one notable difference between human and mouse appears to be the absence of BCL11A in the majority of human myeloid cell lines (Figure 2) even though Bcl11a was initially described in myeloid leukemias arisen in the mouse BXH2 recombinant inbred strain [6,7]. With few exceptions, BCL11A-XL protein is expressed in pre-GC and GC-derived B lymphoid cell lines, but is low to negative in cell lines representative of late-GC or post-GC stages of B cell development. The GC-derived Burkitt lymphomas are phenotypically diverse and the expression of BCL11A-XL does not correlate with the presence or absence of Epstein-Barr virus; however, as a trend, those Burkitt's which display a type III viral latency (a lymphoblastoid phenotype) tend to be negative for BCL11A-XL. This trend is supported indirectly by the observation that type III lines tend conversely to be positive for the putative repressor of BCL11A, namely, Blimp-1 [20]. Among the post-GC lines, the majority of multiple myeloma and Hodgkin's lymphoma samples were negative for BCL11A-XL protein. This agrees well with published microarray data [21-24]. The probes used in microarray experiments further indicate that all BCL11A transcripts are coordinately down-regulated in these post-GC cell lines.

Our related studies using BCL11A/123 for immunostaining [14] indicate that XL is expressed at low to undetectable levels in most normal adult tissues but has highest expression in hematopoietic cells. The presence of XL in mantle zone and GC subsets while absent in normal plasma cells is consistent with the Western data of representative cell lines presented here.

The expression pattern of BCL11A-XL among B-cell lines suggests that its down-regulation might be a requirement for plasmacytic differentiation. This observation is supported by previous gene expression profiling studies where BCL11A-XL was identified as a possible target gene repressed by Blimp-1, a master regulator of plasma cell development [21,24]. In support of this observation, we have identified a subset of putative BCL11A target genes which overlap with confirmed targets of Blimp-1 in plasma cells (G.C.I., P.W.T., and A.L.S, unpublished observations).

### BCL11A-XL binds specifically to DNA and interacts with BCL6

We determined by reiterative oligonucleotide selection that in vitro BCL11A-XL binds to the core consensus 5'-C-C-C/T-A/G-C-3' (Figure 5E). This sequence differs from the consensus binding sequence (5'-GGCCGGAGG-3') determined in a similar manner for the mouse BCL11A-L equivalent, Evi9a/CTIP1 [11]. We were unable to confirm specific binding to this sequence for either the human L form or for the XL and S isoforms (data not shown). The basis for this is unclear. We noticed that nearly 25% of our individual BCL11A-XL CASTing sequences constituted E-boxes (CAXXTG) or contained isolated E-boxes nearby. One particular E-box, CACCTG, can be found immediately upstream of numerous mature B-cell signature genes (e.g., CD19, CD21, and CD37) that are modulated, like BCL11A, by the induction or over-expression of Blimp-1 [21,24].

As shown previously for the mouse L form equivalent [7], human XL and L also associate physically with the protein product of the BCL6 proto-oncogene (Figures 4A and 4B). The association of BCL11A and BCL6 also has been recently confirmed by mass spectroscopy [25]. BCL6 encodes a multiple zinc finger-containing, sequence-specific DNA binding transcriptional repressor that is frequently deregulated by translocation into *IGH *or into (at least) 20 other non-*IGH *loci in FL and DLBCL [26]. BCL6 targets include genes involved in B cell activation, B cell differentiation (e.g., Blimp-1), inflammation, and cell cycle control [27,28].

Since BCL11A and BCL6 are both proto-oncogenes with overlapping expression patterns, a clear biological significance could be envisioned for the heterodimerization of their protein products. Therefore, we were surprised at our inability to demonstrate robust reciprocal effects of BCL6 and BCL11A in transcription assays, whether we used B-cell lines negative (M12.4) or positive (BCL1, DT40) for endogenous BCL11A (Figure 5A–C), even though BCL6/BCL11A-XL association *in vitro *appears to alter DNA binding of either factor to its specific target sequence (Figures 5E and 5F). There are several technical explanations. Nuclear matrix-associated proteins such as BCL11A-XL and BCL6 have been shown to most efficiently transactivate reporters that are integrated within chromatin – a condition not achieved in our transient assays. Alternatively, BCL11A isoform interactions in some cells might be strongly biased toward the homomeric, leaving insufficient opportunity for BCL6 to form heteromeric interactions *in vivo *(albeit the heterodimer can be observed *in vitro *or after lysis of intact cells). An explanation we favor is that the BCL6/BCL11A-XL interaction is merely indirect; ie, both proteins are sequestered proximally in the same subnuclear compartment and both associate with an intermediary protein complex. One such intermediary may be the Mi-2/NuRD co-repressor complex. Recent data demonstrate the interaction of NuRD (specifically the MTA subunits) with several transcription factors, including p53, BCL6, BCL11B, and BCL11A [29-31]. Although they appear to direct transcription independently, BCL6 and BCL11A may antagonize one another at the post-transcriptional level to contribute to the germinal center reaction through their interactions with the Mi-2/NuRD co-repressor complex.

### BCL11A and BCL6 reside in nuclear paraspeckles

BCL6 was initially reported to colocalize with mouse Evi9a in nuclear dots of unknown identity [7]. We have determined by colocalization of documented markers that the BCL11A-XL and BCL6 particles are paraspeckles. Paraspeckles are nuclear aggregates previously shown to contain hnRNA and three paraspeckle-specific (PSP) proteins [32]. Two of these (PSP1 and nonO) were shown to colocalize with BCL11A-XL (Figure 6E), and nonO can form an immunoprecipitable complex with XL (data not shown). Paraspeckles were thought to function exclusively in RNA metabolism. Our data challenge that contention and assign BCL11A and BCL6 as the first DNA-binding transcription factors to reside in this compartment. The subnuclear localization of BCL11A-XL in paraspeckles suggests that it may be involved in some topological aspect of transcription. Indeed, as shown in Figures 6D and 6F, transcription inhibitors and treatment with DNase (but not RNase) can significantly alter the subnuclear partitioning of BCL11A-XL. This was similarly documented for PSP1 and nonO and referred to as peri-nucleolar capping [33]. DNAse-mediated conversion to a diffused pattern indicated that paraspeckle structure is intimately associated with DNA structural integrity, such as provided by the nuclear matrix and its resident proteins, such as BCL11A-XL, BCL6, PSP1, and nonO.

Our subcellular localization studies may provide an alternative and novel explanation for the lack of functional consequences of BCL6/BCL11A-XL interaction. Contents of paraspeckles have been shown to translocate into nucleoli under conditions of cellular stress [32]. Accordingly, we observed under transcriptional starvation and following cell damage with etoposide that BCL11A-XL and BCL6 re-localized to nucleoli in U2OS osteosarcoma cells (data not shown). Often these nuclei showed a "donut" type pattern, in which BCL6 accumulated along the rim, and BCL11A-XL within the center. This pattern is strikingly similar to the "BCL6 dots" observed by electron microscopy [34] and suggests that, under conditions of stress/DNA damage, XL and BCL6 collaboration *in vivo *may be inhibited because they are sequestered in discrete regions of the nuclear dot and incapable of functional association. Such conditions might exist in GC B cell nuclei as they undergo extensive double-stranded DNA damage during the SHM process.

### BCL11A isoform complexity and hematopoietic malignancies

The N-terminus and C2HC zinc-finger are highly conserved across evolution within BCL11A (Figure 1) and among at least four other genes (EHZF, FOG1, FOG2, and BCL11B) such as to define a superfamily [35]. All five genes are essential to myeloid (EHZF), megakaryocytic (FOG1 and FOG2), T-lymphoid (BCL11B) and B-lymphoid (BCL11A) development; three (BCL11A and B, EHZF) have been implicated in hematological malignancy [10,36-38]. In transient transfection assays, BCL11A isoforms act as repressors (Figure 5A–C). However, consistent with what has been observed for the FOG superfamily members [37,39], homo- and heterodimerization of BCL11A likely occurs via the conserved C2HC zinc finger, and protein dimers might activate or repress transcription depending on context. Like the FOG genes, post-translational modifications of BCL11A or associated corepressors (e.g., Mi-2/NuRD) might regulate its activity as either a repressor or activator of transcription. We anticipate that some BCL11A targets will be shared and others will be isoform-specific.

The first 12 amino acids of the conserved N-terminus, when tethered to GAL4DBD, are both necessary and sufficient to mediate repression of transcription [40]. This N-terminal superfamily repressor domain provides a rationale for our observation that the cytoplasmic S form can repress transcription. These data are consistent with studies of Evi9/CTIP1, indicating that a trans-repression domain resides within the shared N-terminal region of all isoforms [11,13]. Although each of the four BCL11A isoforms can homodimerize with itself or heterodimerize with any of the other isoforms, only the nuclear forms (XL and L) interact with BCL6. A dramatic consequence of heterodimerization of the cytoplasmic isoforms with the nuclear isoforms is their nuclear translocation (shown in Figure 7 for S) to nuclear paraspeckles when heterodimerized with either XL or L forms. The data indicate that the interactions and probably the ratios among BCL11A isoforms and between BCL11A and BCL6 will be important in controlling their functions.

## Conclusion

BCL11A is one representative of a superfamily of transcription factors known to be involved in normal as well as malignant hematopoetic development. BCL11A can modulate transcription activity in vitro but in a way that is likely to be context dependent in vivo since we have shown that BCL11A can interact with other proteins (namely, the BCL6 proto-oncogene) and that BCL11A exhibits different subcellular and subnuclear compartmentalization according to which of the various isoforms is examined. Here we have focused attention on the novel XL isoform which had heretofore been undescribed. We have shown that BCL11A-XL is a DNA-sequence-specific transcriptional repressor in vitro, which, like the transcription factors BCL6 and nonO, localizes to nuclear paraspeckles in vivo. Because of the ability of BCL11A-XL to associate with itself as well as with other BCL11A isoforms, future studies of BCL11A will require a careful and thorough description of all the alternative splice variants produced at this locus.

## Methods

### Plasmids

HA- and FLAG-N-terminally-tagged BCL11A-XL, BCL11A-L, and BCL11A-S were constructed from human cDNAs into the pCR3.1 mammalian expression vector (Invitrogen) using PCR with the same forward primer but different reverse primers for each isoform. BCL11A-XL, BCL11A-L and BCL11A-S were amplified by PCR with the forward primer 5-pCR3.1-BCL11(SalI): 5'-CTACAACAGGTCGACATGTCTCGCCGCAAG-3', and the following reverse primers for each of the isoforms, XL, L, and S:

BCL11A-XL:

5'ATACCTCTATTCAGTTTTTATATCATTATTCAACACTCGATCACTGTGCCATTTTTTCATGTGTTTCTCCAGGGTACTGTACACGTCA-3'

BCL11A-L:

5'-CATAGT-AGCCTGCAGGTGTCGCTGCGTCTG-3'

BCL11A-S:

5'-CATATTAGCCTGCAGCGCGGGGTCAGGGGA-3'.

N-terminal, GFP-fusions were derived from these clones following the same PCR strategy, and the amplified cDNAs were cloned into SmaI and BamH1 sites of pEGFP-C1. BCL11A-XL, BCL11A-L and BCL11A-S were amplified by PCR with the forward primer 5-pEGFP-BCL11(SalI): 5'-CTACAACAGGTCGACATGTCTCGCCGCAAG-3' and the following reverse primers:

BCL11A-XL:

5'-CATCACAGTGGATCCATACCTCTATTCAGT-3'

BCL11A-L:

5'-CATATTATCGGATCCGTGTCGCTGCGTCTG-3'

BCL11A-S:

5'-CATATTATCGGATCCCGCGGGGTCAGGGGA-3'

GAL4 DNA binding domain (DBD) N-terminal fusions of each isoform utilized a similar strategy with a common forward primer used to generate the construct for all three isoforms, and amplified cDNAs were cloned into BamHI and KpnI sites of pBind (Promega). All isoforms were amplified using the same forward primer 5-GAL4DBD-BCL11(BamHI): 5'-ATAGTCGGATCCGCATGTCTCGCCGCAAG-3' and the following reverse primers:

BCL11A-XL:

5'-CGCGACGCGGGTACCTATCGAATTCTTCCA-3'

BCL11A-L:

5'-CATCACAGTGGTACCGTGTCGCTGCGTCTG-3'

BCL11A-S:

5'-CATATCAGCGGTACCCGCGGGGTCAGGGGA-3'

A full-length BCL6 mammalian expression vector, pCMV-BCL6, was kindly provided by Dr. Riccardo Dalla-Favera.

### Cell cultures

COS-7, NIH-3T3, HeLa and HEK293 cells were maintained in DMEM (Gibco-BRL) with 10% FBS (HyClone). All B-cell lines (BJAB, Namalwa, Granta-452, DT-40, RAJI, RAMOS) were maintained in RPMI-1640 media (Gibco-BRL) with 10% FBS. The BCL11A/123 hybridoma producing anti-BCL11A-XL antibody was cultured as described [14]. Derivation and characterization of the other cell lines used in this study (Figure 2) may be obtained upon request (MJSD).

### Protein fractionation

Nuclear matrix fractionation was carried out according to the method of Reyes et al [15]. Ten μl of 5 × SDS sample buffer was added to 40 μl of a fractionated lysate (cytoplasm [C], nucleoplasm [NP], chromatin [CH], or nuclear matrix [NM]) and the mixture was boiled for 5 min. Twenty μl of extract in 1 × SDS sample buffer was loaded for gel electrophoresis.

### Western blot analysis

Cells were suspended in RIPA lysis buffer (50 mM Tris-Cl, pH 8.0; 150 mM NaCl; 2 mM EDTA; 1% NP40; 0.1% SDS; 0.5% sodium deoxycholate; Roche complete protease inhibitor cocktail) on ice 10 min, then cleared by centrifugation. Lysates were separated by SDS-PAGE and transferred to nitrocellulose membranes (Protran BA, Schleicher and Schuell). Primary antibodies: mouse anti-HA monoclonal antibody (Babco), 1:1000; mouse anti-FLAG M2 monoclonal antibody (Sigma) 1:10000; BCL11A/123 as straight hybridoma supernatant; clone N3 anti-BCL6 rabbit polyclonal antibody (Santa Cruz Biotechnology) 1:1000. Primary antibodies were incubated 3 hr at room temperature or at 4°C overnight, washed (PBS with 0.05% Tween-20) 3 times, then incubated with secondary antibodies (Horseradish peroxidase-conjugated goat anti-mouse or goat anti-rabbit [Amersham Pharmacia Biotech]) at 1:8000 for 1 hr at room temperature. Blots were developed using the ECL Western blotting detection reagent (Amersham).

### Fluorescence imaging

#### BCL11A and BCL6 colocalization experiments in transfectants (Figure 6A)

COS-7 cells were co-transfected with 1 μg of pCMV-FLAG-BCL11A-XL (*left panel*) or pEGFP-BCL11A (*right panel*) and 1 μg of pCMV-FLAG-BCL6 using Fugene 6 (Roche) according to the manufacturer's instructions. 48 hr post-transfection, cells were fixed (4% paraformaldehyde in PBS for 10 min), washed, and then permeabilized (0.5% Triton X-100 in PBS for 15 min). Primary antibodies were incubated 45 minutes: BCL11A/123 straight supernatant plus N3 anti-BCL6 1:400 in 2% BSA (*left panel*); M2 anti-FLAG mAb at 1:1000 (*right panel*). After washing, secondary antibodies (Molecular Probes) were diluted 1:400 in 2% BSA and incubated 10–15 minutes (goat anti-mouse Alexa 488 plus goat anti-rabbit Alexa 594; or rabbit anti-mouse Alexa 594, all at 1:400). Slides were washed twice 30–45 min, and mounted with VectaStain (Vector Labs) containing Hoescht 33342 (Sigma) at 1:1000. Cells were viewed by sequential confocal laser scanning microscopy (Leica SP2 AOBS).

#### Endogenous BCL11A and BCL6 colocalization experiments in B cells (Figure 6B)

BJAB or Ramos cells were washed, fixed, permeabilized, blocked with 10% goat serum in 2% BSA for 30 min before a 1 hr incubation with BCL11A/123 and N3 anti-BCL6 (as above). Secondary antibodies were incubated as above (goat anti-mouse Alexa 488, BCL11A; goat anti-rabbit Alexa 594, BCL6).

#### BCL11A colocalization experiments with SC35, PSP1, and NonO/p54nrb (Figures 6C and 6E)

COS-7 or HEK293 were transiently transfected with 1 μg of pEGFP-BCL11A isoforms as described above. Cells were fixed, permeabilized, and incubated with blocking buffer containing 3% bovine serum albumin and 0.2% gelatin in PBS for 15 min, and then incubated with primary antibodies: Mouse anti-SC35, 1:2000 (Sigma); mouse anti-nonO, 1:200 (kind gift of R.B. Moreland); rabbit anti-PSP1, 1:1000 (kind gift of A. Fox and A.I. Lamond). Goat anti-mouse Cy3 (1:200, Amersham) and goat anti-rabbit Cy2 (1:200, Amersham) were then used as secondary antibodies

### Immunoprecipitation

For *in vitro *co-immunoprecipitations, Gal4DBD-BCL11A isoforms and ^35^S-labeled HA-BCL11A isoforms generated by *in vitro *translation were incubated in the presence or the absence of mouse anti-Gal4-DBD monoclonal antibody (Sigma) at 1:100 dilution in IP buffer (50 mM Tris-Cl, pH 7.5, 150 mM NaCl, 5 mM MgCl_2_, 1 mM EDTA, 0.25% NP-40, 1 mM DTT, 1 mM PMSF) for 1 hr at 4°C. Protein A agarose (Sigma)/IP slurry was added and incubated 1 hr at 4°C. The mixture was centrifuged and pellets were washed extensively with IP buffer prior to separation on 8% SDS-PAGE and visualization by phosphorimaging.

For *in vivo *co-immunoprecipitation of recombinant proteins, HEK293 cells co-transfected with pCR3.1-HA-BCL11A and pCMV-FLAG-BCL6 were washed, lysed in cold RIPA buffer, pre-cleared with protein A beads for 30 min at 4°C, and then incubated with anti-FLAG at 1:100 dilution for 1 hr at 4°C. IPs were collected on protein A beads as above, were washed in RIPA buffer, then examined by SDS-PAGE/Western blotting using anti-HA antibody.

For co-immunoprecipitation of endogenous BCL11A and BCL6, 10 μl of N3 anti-BCL6 was added to the RIPA lysate of 1 × 10^7 ^Raji cells and incubated overnight at 4°C. Rabbit polyclonal anti-CRM-1 (Santa Cruz Biotechnology) was used as negative control. Complexes were pulled down using Protein A beads, washed repeatedly with RIPA lysis buffer, then eluted and resolved by SDS-PAGE, followed by Western blotting the C-terminus of BCL11A (AbCam ab19489, 1:1000 dilution in 5% milk).

### Luciferase assays

HEK293, seeded at 2 × 10 ^5 ^cells and cultured for 18 hr, were transiently co-transfected with 1 μg of pG5*luc *luciferase reporter construct (Promega), and 1 μg of Gal4DBD or Gal4DBD-BCL11A isoform constructs using Fugene 6. Luciferase activity was measured 48 hr post-transfection using the Dual-Luciferase Reporter Assay System (Promega) according to the manufacturer's directions. Values were expressed as percent of those obtained using the reporter construct and Gal4DBD alone after normalization of transfection efficiency for the activity of *Renilla *luciferase encoded in the Gal4DBD vector.

For B-cell lines (BCL1, M12.4 and DT-40), cells were transiently co-transfected with 10 μg of pG5*luc *and 30 μg of Gal4DBD or Gal4DBD-BCL11A isoform constructs by electroporation (BioRad) under conditions empirically determined as optimal for each cell line. Luciferase activity was measured as described above.

For co-transfection of BCL11A and BCL6, HEK293 cells were transiently co-transfected with 1 μg of pG5*luc*, 1 μg of Gal4DBD or Gal4DBD-BCL11A isoform constructs and 1 μg of pCMV-FLAG-BCL6 by Fugene 6 reagent. DT-40 B cells were transiently co-transfected with 20 μg of Gal4DBD or Gal4DBD-BCL11A isoform constructs and 20 μg of pCMV-FLAG-BCL6 or irrelevant DNA pCEP4. The luciferase activity was measured as described above. For luciferase assays of BCL6 activity, we employed a luciferase reporter in which the minimal SV40 promoter is appended upstream with 5×-BCL6 binding sites in tandem (kind gift of Vivian Bardwell) and pRL-TK (Promega) as a transfection efficiency control.

### Cyclic Amplification and Selection of Target (CASTing) analysis

CASTing was carried out as previously described [16] with minor modifications. A random oligonucleotide pool of 76 nucleotides containing unique flanking sequence (5'-CAGGTCAGTTCAGCGGATCCTGTCG(A/G/C/T)26GAGGCGAATTCAGTGCAA-CTGCAGC-3') was rendered double-stranded by second strand synthesis. Two ng of N-terminal HA tagged BCL11A-XL *in vitro *translation product and 0.5 ng of anti-HA antibody 9E10 were incubated in Binding Buffer (20 mM HEPES pH 7.9, 40 mM KCl, 6 mM MgCl_2_, 1 mM DTT, 0.1% NP40, 3 mg/ml acetylated BSA, 10% glycerol, 2% Ficoll, 50 mg/ml sonicated salmon sperm DNA, 100 mg/ml PMSF, 1 mg/ml aprotinin) on ice for 30 min. Recovered DNA was PCR amplified using 5'-GCTGCAGTTGCACTGA-ATTCGCCTC-3', and 5'-CAGGTCAGTTCAGCGGATCCTGTCG-3'. Five rounds of selection and amplification were carried out and the final PCR products were subcloned and sequenced.

### Electrophoretic mobility-shift assay (EMSA)

An oligonucleotide containing four BCL11A-XL consensus binding sites 5'GCACTCCCACGCCTCACTCCCACGCCTCACTCCCACGCCTCCCACCCCCGCC TCCCACCCCCGCCT-3' and its complement were used as probe for Figure 5E. For Figure 5F, the tandem BCL6 binding site repeat was isolated as a fragment from 5XBCL6-SV40-Luc and used as probe. Binding reactions used Binding Buffer as above.

## Competing interests

The author(s) declare there are no competing interests.

## Authors' contributions

All authors listed participated in the design and/or execution of experiments. H.L. contributed to the design and execution of the experiments shown in Figures 4A, 4C, 5A-F, 6A, 6D, 6F, and 7; G.C.I. to Figures 1, 3C, 6A, and 6B; J.K.W. to Figures 5A–C, and 6A–B; T.N. to Figures 6C–E; L.P. to Figure 1 and the content of the Discussion; B.S.L. to Figure 3C; K.P and A.H.B. L.S. to Figure 2; M.J.S.D. to Figures 2 and 6A; A.L.S. and L.M.S. to Figure 5F; and C.D. to Figures 3, 4B, 5E, and 5F. G.C.I. and P.W.T. wrote the paper, and all authors contributed to the final copy of the paper. All authors have read and approved the final manuscript.
